# Evaluation of antifungal effect and toxicity of xanthyletin and two bacterial metabolites against Thai isolates of *Pythium insidiosum*

**DOI:** 10.1038/s41598-020-61271-0

**Published:** 2020-03-11

**Authors:** Kittiya Wittayapipath, Chavi Yenjai, Chularut Prariyachatigul, Petr Hamal

**Affiliations:** 1 Medical Science Program, Graduate School, Faculty of Associated Medical Sciences, Centre for Research and Development of Medical Diagnosis Laboratories, Khon Kaen University, Khon Kaen, Thailand; 2 Department of Chemistry, Faculty of Science, Khon Kaen University, Khon Kaen, Thailand; 3 Department of Clinical Microbiology, Faculty of Associated Medical Sciences, Centre for Research and Development of Medical Diagnosis Laboratories, Khon Kaen University, Khon Kaen, Thailand; 4 Department of Microbiology, Faculty of Medicine and Dentistry, Palacky University Olomouc, Hnevotinska 3, 77515 Olomouc, Czech Republic

**Keywords:** Drug safety, Antifungal agents, Cellular microbiology, Fungal infection

## Abstract

Pythiosis is a harmful disease caused by *Pythium insidiosum*, an aquatic oomycete. Therapeutic protocols based on antifungal drugs are often ineffective because the cytoplasmic membrane of *P. insidiosum* does not contain ergosterol. Therefore, the treatment of pythiosis is still challenging, particularly making use of natural products and secondary metabolites from bacteria. In this study, xanthyletin and substances obtained from *Pseudomonas stutzeri* ST1302 and *Klebsiella pneumoniae* ST2501 exhibited anti-*P. insidiosum* activity and, moreover, xanthyletin was non-toxic against human cell lines. The hyphae of *P. insidiosum* treated with these three substances exhibited lysis holes on a rough surface and release of anamorphic material. Therefore, xanthyletin could be considered a promising alternative agent for treating cutaneous pythiosis in the near future.

## Introduction

*Pythium insidiosum* is an aquatic oomycete, or fungus-like microorganism. Its morphology is similar to that of filamentous fungi but a phylogenetic analysis of this pathogen has shown a closely related to diatoms and algae than to true fungi^1^. *P. insidiosum* can infect both humans and animals through the eyes or skin wounds, causing pythiosis, an endemic disease with high morbidity and mortality rates^2,3^. Human pythiosis manifests clinically as cutaneous and subcutaneous, vascular, ocular or disseminated infection. The treatment includes antifungal agents, radical surgery and immunotherapy. However, these options have not always been successful and generally accepted therapeutic protocols have not yet appeared^2,4^. Most of the common antifungal drugs are ineffective because the cytoplasmic membrane of *P. insidiosum* does not contain ergosterol or this pathogen possesses an incomplete ergosterol biosynthetic pathway^5^. Therefore, the treatment of pythiosis is still challenging. Previous studies demonstrated that natural compounds from plants and secondary metabolites from microorganisms had relatively good *in vitro* antimicrobial activity against *P. insidiosum*^6,7^. Xanthyletin, a natural compound of green plants, is classified in a group of pyranocoumarins, coumarin derivatives. Antifungal activity of xanthyletin against *Candida albicans*, *Aspergillus fumigatus* and *Fusarium solani* as well as its anti-*P. insidiosum* effect have been reported^6,8^. Besides, its anticancer and antibacterial activity were also described^9^. Bacterial species belonging to the genus *Pseudomonas*, especially those isolated from the environment, are often used as biocontrol agents in economic crops since the pathogen excretes substances with antibacterial and antifungal activity^10^. Moreover, microorganisms of the genus *Klebsiella* produce secondary metabolites with antibacterial and antifungal activity as well^11^. An *in vitro* study of secondary metabolites excreted from bacterial environmental strains included *Pseudomonas stutzeri* and *Klebsiella pneumoniae* from 16 strains that expressed anti-*P. insidiosum* activity^7^. However, the mechanisms of action of both xanthyletin and secondary metabolites from bacteria with anti-*P. insidiosum* activity remain unclear. Fibroblasts are one of the most common cell types, widely present in many structures, particularly in connective tissue and predominantly in the human dermis. Fibroblasts are of mesenchymal origin, exhibiting a spindle or stellate shape morphology. These cells play an important role in the cutaneous wound healing process^12,13^. As one of the clinical manifestations of human pythiosis is cutaneous and subcutaneous infections, toxicity testing of substances with anti-*P. insidiosum* effects on these cell lines are important in terms of their safety in the treatment of pythiosis. Therefore, this study evaluated the anti-*P. insidiosum* effect of xanthyletin and secondary metabolites from *P. stutzeri* and *K. pneumoniae* on *P. insidiosum* by broth dilution susceptibility testing and scanning electron microscopy (SEM). The toxicity of these substances to the cell lines prepared from normal human dermal fibroblast (NHDF) cells was also assessed.

## Results

### Crude extracts and fractions from *P. stutzeri* ST1302 and *K. pneumoniae* ST2501 and their anti-*P. insidiosum* activity

**Figure 1:**
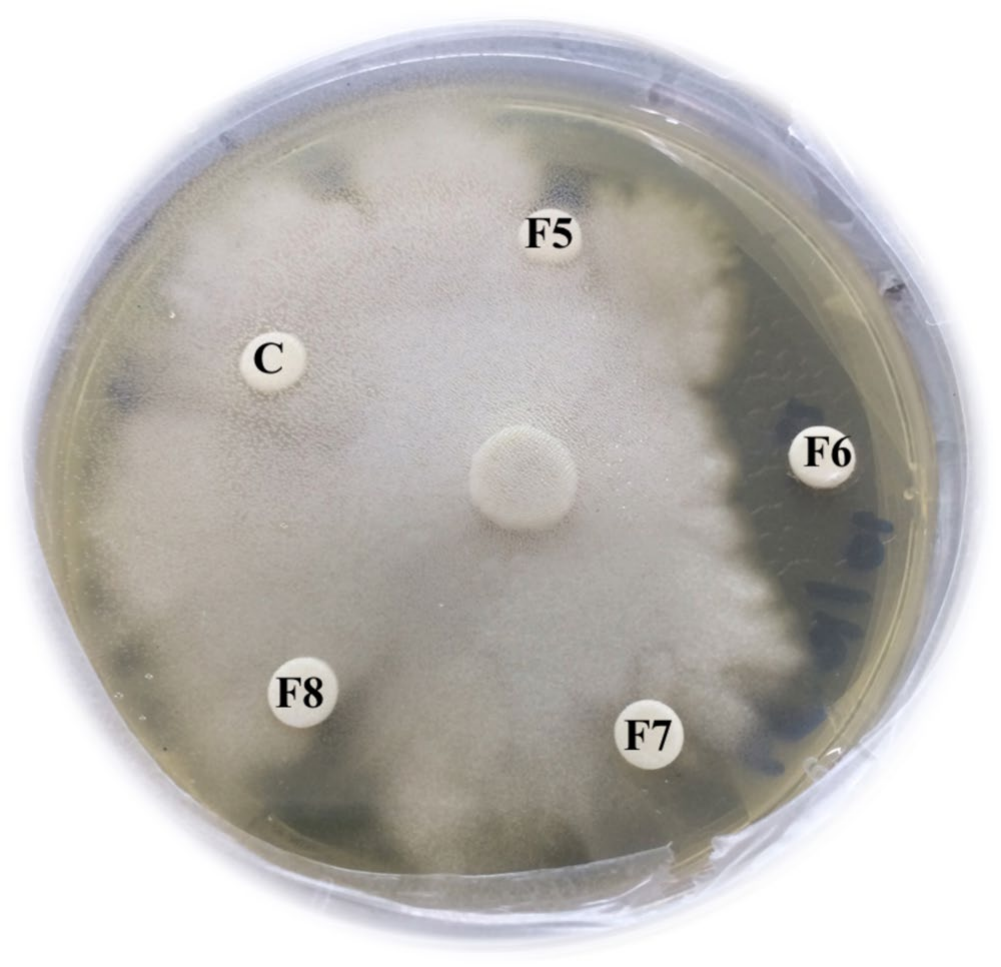
Screening for anti-*Pythium insidiosum* activity of fractions no. 5, 6, 7 and 8 of the crude extract from *Pseudomonas stutzeri* ST1302 by the disc diffusion method. The inhibition zone was found around fraction no. 6 which means it had anti-*P. insidiosum* activity. C = control disc (dichloromethane), F = fraction number.

Crude extracts from *P. stutzeri* ST1302 (brown liquid) and *K. pneumoniae* ST2501 (dark brown liquid) showed anti-*P. insidiosum* activity as screened by the disc diffusion method. After that, the crude extracts were fractionated by activity-guided separation liquid column chromatography. The crude extracts from *K. pneumoniae* ST2501 were divided into 5 fractions; fraction number 1 was brown semisolid, fraction number 2 was brown oil, fraction number 3 was brown solid, fraction number 4 was black oil and fraction number 5 was black semisolid. The crude extracts from *P. stutzeri* ST1302 were divided into 14 fractions; fraction no. 1 was yellow oil, fraction no. 2 was orange oil, fractions no. 3, 4, 5, 7 and 9 were deep yellow solid, fraction no. 6 was yellow solid, fractions no. 8, 12, 13 and 14 were brown oil, and fractions no. 10 and 11 were white solid. Fractions no. 1 to 4 of the crude extracts from *K. pneumoniae* ST2501 and fractions no. 3, 4, 6, 9 and 12 of the crude extracts from *P. stutzeri* ST1302 exhibited anti-*P. insidiosum* activity (Table 1). The strongest anti-*P. insidiosum* activity (the largest inhibition zone) was exhibited by fraction no. 6 of the crude extract from *P. stutzeri* (Fig. 1) eluted with 20% methanol in dichloromethane and fraction no. 1 of the crude extract from *K. pneumoniae* eluted with 100% dichloromethane. For further testing, only two fractions mentioned above with the strongest anti-*P. insidiosum* activity together with xanthyletin were chosen.

**Table 1:** The characteristics of fractions of the crude extracts from bacteria, screening for anti-*Pythium insidiosum* activity and the minimum fungicidal concentrations of anti-*P. insidiosum* substances against 11 *P. insidiosum* strains isolated from pythiosis patients in Thailand.

Substances	Fraction number	Characteristics	Screening for anti-*P. insidiosum* activity by the disc diffusion method	Minimum fungicidal concentrations (mg/mL) against *P. insidiosum* strains										Disseminated MCC29
				Vascular						Ocular				
				SIMI-2989–42	SIMI-7873	SIMI-7874	SIMI-8659	SIMI-8727	MCC5	SIMI-6666	SIMI-18093	SIMI-322–37	SIMI-9743	
The crude extract from *Klebsiella pneumoniae* ST2501	1	Brow semisolid	Active	3.125	3.125	3.125	1.563	1.563	1.563	3.125	1.563	1.563	1.563	1.563
	2	Brown oil	Active	—	—	—	—	—	—	—	—	—	—	—
	3	Brown solid	Active	—	—	—	—	—	—	—	—	—	—	—
	4	Black oil	Active	—	—	—	—	—	—	—	—	—	—	—
	5	Black semisolid	Inactive	—	—	—	—	—	—	—	—	—	—	—
The crude extract from *Pseudomonas stutzeri* ST1302	1	Yellow oil	Inactive	—	—	—	—	—	—	—	—	—	—	—
	2	Orange oil	Inactive	—	—	—	—	—	—	—	—	—	—	—
	3	Deep yellow solid	Active	—	—	—	—	—	—	—	—	—	—	—
	4	Deep yellow solid	Active	—	—	—	—	—	—	—	—	—	—	—
	5	Deep yellow solid	Inactive	—	—	—	—	—	—	—	—	—	—	—
	6	Yellow solid	Active	3.125	3.125	3.125	3.125	3.125	3.125	3.125	3.125	3.125	3.125	3.125
	7	Deep yellow solid	Inactive	—	—	—	—	—	—	—	—	—	—	—
	8	Brown oil	Inactive	—	—	—	—	—	—	—	—	—	—	—
	9	Deep yellow solid	Active	—	—	—	—	—	—	—	—	—	—	—
	10	White solid	Inactive	—	—	—	—	—	—	—	—	—	—	—
	11	White solid	Inactive	—	—	—	—	—	—	—	—	—	—	—
	12	Brown oil	Active	—	—	—	—	—	—	—	—	—	—	—
	13	Brown oil	Inactive	—	—	—	—	—	—	—	—	—	—	—
	14	Brown oil	Inactive	—	—	—	—	—	—	—	—	—	—	—
Xanthyletin	–	White powder	Active	0.003	0.003	0.003	0.003	0.003	0.003	0.003	0.003	0.003	0.003	0.003

### Minimum fungicidal concentrations (MFCs) determined by broth dilution susceptibility testing

The MFCs of xanthyletin, fraction no. 1 of the crude extract from *K. pneumoniae* ST2501, and fraction no. 6 of the crude extract from *P. stutzeri* ST1302 against 11 strains of *P. insidiosum* isolated from human pythiosis in Thailand were 0.003, 1.563–3.125, and 3.125 mg/mL, respectively (Table 1).

### Hyphal morphology evaluated by scanning electron microscopy

**Figure 2:**
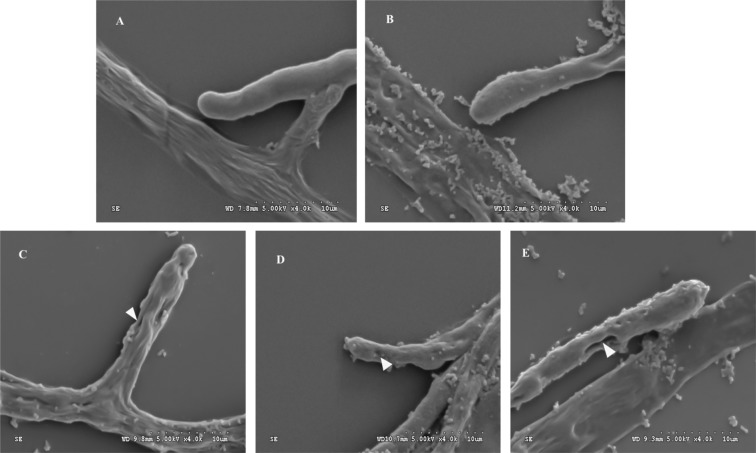
Scanning electron microscopy of *Pythium insidiosum*. (**A**) Negative control (*P. insidiosum* survival): cylindrical and a smooth surface of hyphae. (**B**) Positive control (*P. insidiosum* treated by thimerosal): a rough surface and high amount of released anamorphic material. (**C**–**E**) *P. insidiosum* treated with three anti-*P. insidiosum* substances; all pathogens exhibited lysis holes (head arrows) on a rough surface and released anamorphic material: (**C**) effect of xanthyletin, (**D**) effect of fraction no. 6 of the crude extract from *Pseudomonas stutzeri* ST1302, (**E**) effect of fraction no. 1 of the crude extract from *Klebsiella pneumoniae* ST2501.

Figure 2 shows morphological hyphae of *P. insidiosum* (negative control; *P. insidiosum* survival) and hyphae changes when *P. insidiosum* was treated with thimerosal (positive control; *P. insidiosum* death) and treated with three anti-*P. insidiosum* substances. The negative control showed cylindrical hyphae with a smooth surface (Fig. 2A). Hyphae from the positive control were with a rough surface and high amount of anamorphic material released from the organism (Fig. 2B). Morphology of hyphae treated with three anti-*P. insidiosum* substances including xanthyletin (Fig. 2C), fraction no. 6 of the crude extract from *P. stutzeri* ST1302 (Fig. 2D) and fraction no. 1 of the crude extract from *K. pneumoniae* ST2501 (Fig. 2E) exhibited lysis holes on a rough surface and anamorphic material released from hyphae.

### Cytotoxicity assay

**Figure 3:**
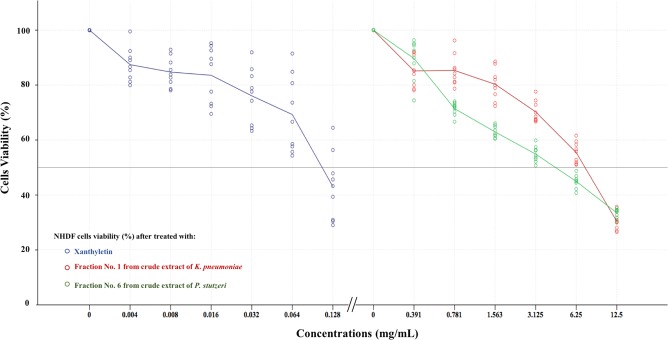
Survival rates of normal human dermal fibroblast cells against various concentrations of xanthyletin, fraction no. 1 of the crude extract from *Klebsiella pneumoniae* ST2501 and fraction no. 6 of the crude extract from *Pseudomonas stutzeri* ST1302 after 24 h.

The toxicity testing of xanthyletin and extracts from *P. stutzeri* ST1302 and *K. pneumoniae* ST2501 covered the whole ranges of dilutions tested for MFC determination by the broth dilution method. The effect of anti-*P. insidiosum* substances on NHDF cells was dose-dependent. The IC50 of xanthyletin, fraction no. 6 of the crude extract from *P. stutzeri* ST1302 and fraction no. 1 of the crude extract from *K. pneumoniae* ST2501 were 0.11, 4.69, and 7.81 mg/mL, respectively (Fig. 3). Further, at the MFC of xanthyletin, NHDF cells showed more than 90% viability, compared to only 55% and 70% viability in case of the MFCs of the tested fractions from *P. stutzeri* and *K. pneumoniae*, respectively.

## Discussion

It is well known that the therapy of pythiosis has posed a challenge in recent decades as the current treatment options are highly variable and often lead to failure^14^. Therefore, many studies have searched for antimicrobial agents, natural compounds or secondary metabolites from microorganisms against *P. insidiosum* and results of *in vitro* testing are quite promising^6,7,15–21^. The present study evaluated antimicrobial effects against *P. insidiosum* of xanthyletin and two fractions of the crude extracts from *P. stutzeri* ST1302 and *K. pneumoniae* ST2501. The MFC results of fraction no. 1 from the crude extract from *K. pneumoniae* against *P. insidiosum* strains SIMI-6666, SIMI-2989–42, SIMI-7873 and SIMI-7874 at higher concentrations than the other strains correlated with the previous study^21^. Xanthyletin showed the best anti-*P. insidiosum* activity and the MFC of 0.003 mg/mL was well correlated with compound 5 from *Scaevola taccada* fruits exhibiting good anti-*P. insidiosum* activity (the minimum inhibitory concentration 0.005 mg/mL)^6^. SEM analysis of *P. insidiosum* treated with anti-*P. insidiosum* substances revealed lysis holes on a rough surface and anamorphic material released from hyphae, suggesting effects on protein composition in the cell wall or cell membrane of this oomycete. Our previous study using Fourier transform infrared spectroscopy found anti-*P. insidiosum* activity of xanthyletin and secondary metabolites from *P. stutzeri* ST1302 and *K. pneumoniae* ST2501, characterized by clearly changed proteins in *P. insidiosum*^21^. Thimerosal is a well-known compound used as a preservative in various cosmetics, vaccines and drug products because of its bactericidal and antifungal properties including anti-*P. insidiosum* activity. However, it is toxic to humans, causing mortality in the fetus, birth defects and neurodevelopmental disorders as evidenced by a case of a pregnant woman who received a vaccine preserved with thimerosal. Besides, cellular apoptosis and abnormal functions of T- and B-cells that affected cytokine production, cell growth and proliferation following chronic exposure to low levels of methyl mercury have also been reported^22,23^. Recently, we used 0.02% (w/v) thimerosal as a positive control because it can kill *P. insidiosum* although this amount is not suitable for use in humans and animals. The mechanism of its action is not fully understood yet and we suspected it to be related to interactions with some proteins in *P. insidiosum* leading to the death of the microorganism^21^. Xanthyletin is a pyranocoumarin that has been shown to be cytotoxic *in vitro* against tumor cells such as Caco-2, HCT-8 and HEp-2 cell lines using an MTT assay^24^. Moreover, xanthyletin showed cytotoxic activity against human cancer HeLa cells without toxicity to normal cells^9^. The NHDF cell culture was selected for cytotoxicity assay as these cells are a common part of the human dermis and toxic effects of drugs on them are undesirable. Moreover, one of the important clinical signs of human pythiosis is skin damage. In the present study, xanthyletin at 0.003 mg/mL showed the best anti-*P. insidiosum* activity and exhibited low toxicity for NHDF cells. However, this study evaluated anti-*P. insidiosum* activity against only 11 strains of *P. insidiosum* isolated from pythiosis patients in Thailand. Therefore, further studies on geographically and genetically diverse *P. insidiosum* strains and anti-*P. insidiosum* activity against pythiosis in animal models should be conducted to confirm the properties of these anti-*P. insidiosum* substances.

## Conclusion

As seen from the evaluation of anti-*P. insidiosum* effects and toxicity of xanthyletin and secondary metabolites from *P. stutzeri* ST1302 and *K. pneumoniae* ST2501, xanthyletin showed excellent anti-*P. insidiosum* activity as well as the lowest toxic effects *in vitro* on NHDF cells. Therefore, it could be considered a promising alternative agent for treating cutaneous pythiosis in the near future.

## Material and Methods

### Microorganisms

*P. insidiosum* isolates (n = 11) from human pythiosis were identified by molecular analysis in a study by Chaiprasert *et al*.^25^. The cultures were maintained on Sabouraud Dextrose Agar (SDA; Himedia) at room temperature and subcultured once a month. According to a study by Thongsri *et al*., they were rechecked by their morphology on the SDA at 25 °C, microscopically by detection of perpendicular sparsely septate hyphae and induced zoospore formation and by PCR identification^7^.

*Pseudomonas stutzeri* ST1302 and *Klebsiella pneumoniae* ST2501 which produced anti-*P. insidiosum* substances were isolated from a water sampling area around Khon Kaen University and identified biochemically using the VITEK 2 system (bioMérieux)^7^. They were stored in 10% Oxoid Skim Milk Powder (Thermo Fisher Scientific) solution with 15% glycerol at −20 °C.

### Substances

#### Xanthyletin (C_14_H_12_O_3_)

It was obtained as a powder (purity 98%, molecular weight 228.24) from ChemFaces (Wuhan).

### Preparation and fractionation of crude extracts from *P. stutzeri* and *K. pneumonia*

The inoculations from *P. stutzeri* ST1302 and *K. pneumoniae* ST2501 were prepared according to McFarland Standard No.1 (3×10^8^ CFU/mL) in saline; 700 µL of this concentration were inoculated into 700 mL of Brain Heart Infusion broth (BHI; Himedia) and incubated in a rotary shaker (200 rpm, 37 °C) for 3 days. Each culture broth was then centrifuged and the supernatant was filtered through a membrane filter (Millipore). The cell-free filtrate was concentrated ten-fold in a rotary evaporator (Rotavapor R-210, Büchi) and mixed three times with a two-fold volume of dichloromethane (Thermo Fisher Scientific) in case of *P. stutzeri* or with ethyl acetate (Thermo Fisher Scientific) in case of *K. pneumoniae*. Both dichloromethane and ethyl acetate layers were dried with anhydrous Na_2_SO_4_ (Merck) and concentrated in a rotary evaporator. Then the crude extracts were fractionated by activity-guided separation liquid column chromatography using a protocol described by Wittayapipath *et al*.^21^.

### Screening for anti-*P. insidiosum* activity by the disc diffusion method

Xanthyletin and the crude extracts from *P. stutzeri* and *K. pneumoniae* were tested for anti-*P. insidiosum* activity using the disc diffusion method. Stock solutions of each testing solution were prepared as 500 mg/mL. Paper discs (6 mm in diameter; Gibthai, Thailand) were placed onto SDA plates with *P. insidiosum* aged 2 days grown on the plates. Twenty microliters of testing solutions were applied to the discs (dichloromethane and ethyl acetate were used as controls) and then the testing plates were stored at room temperature for 2 h in a laminar flow biosafety cabinet to test the solution diffusion. Subsequently, they were incubated at 37 °C for 3, 6 and 9 days and inhibition zones were measured when the growth of *P. insidiosum* reached the control discs^21^.

### Broth dilution susceptibility testing

The method by Trolezi *et al*. was used with some modifications^20^. Briefly, blocks measuring 5 mm in diameter were cut out from SDA plates with *P. insidiosum* cultures with a cork borer. These mycelia-containing blocks were transferred into 1.95 mL of Sabouraud Dextrose Broth (SDB; Himedia) and incubated at 37 °C for 3 days. Dilutions of xanthyletin were prepared from 0.100 to 0.001 mg/mL, dilutions of *P. stutzeri* ST1302 and *K. pneumoniae* ST2501 crude extracts from 12.5 to 0.1 mg/mL by double dilution. Each 50 μL of diluted testing solutions were added to the cultures and incubated at 37 °C for 24 h. A *P. insidiosum* block in 2 mL of SDB was incubated at 37 °C for 4 days as a growth control. After expiration of the incubation time, the *P. insidiosum* agar blocks with mycelium were placed on SDA plates and incubated at 37 °C for 3, 6 and 9 days (depending on the growth speed) to evaluate the hyphal growth and determine the minimum fungicidal concentrations (MFCs). The MFCs were assessed as the lowest concentration of the solution without any apparent hyphal growth. All tests were performed in triplicate.

### Scanning electron microscopy of the hyphal morphology

The protocols described by Trolezi *et al*.^20^ and Mendoza *et al*.^3^ were followed with some modifications. For SEM analysis, hyphae in blocks from each testing solution evaluated as MFCs were selected. *P. insidiosum* hyphal fragments from the growth control in broth dilution susceptibility testing served as a negative control, that is the agent survived. By contrast, *P. insidiosum* hyphal fragments treated with 0.02% (w/v) thimerosal (Sigma-Aldrich) were used as a positive control. Thimerosal is well known as a preservative in various cosmetics, vaccines and drug products to prevent harmful contamination with microorganism. Therefore, thimerosal completely killing *P. insidiosum* was used as a positive control in this study. Hyphal fragments were collected and washed three times in 0.1 M phosphate buffer solution (PBS). Then, they were fixed in 2.5% glutaraldehyde (Merck) in 0.1 M PBS at 4 °C for 1 h and washed in the same buffer. Subsequently, the samples were dehydrated with a graded ethanol series. The Emitech K500X sputter coater (Quorum Technologies) was used for gold sputtering of SEM samples. Finally, the hyphal morphology was evaluated by the S-3000N scanning electron microscope (Hitachi).

### Cytotoxicity assay

Xanthyletin and two anti-*P. insidiosum* substances from *P. stutzeri* ST1302 and *K. pneumoniae* ST2501 were evaluated for cytotoxicity using NHDF cell lines (Promocell). A colorimetric assay with 3-(4,5-dimethylthiazol-2-yl)−2,5-diphenyltetrazolium bromide (MTT) was applied to investigate the cytotoxicity^26,27^. Briefly, NHDF cell lines were counted with the Neubauer hemocytometer using trypan blue solution (10^4^ cells; 100 μL/well). Then, the cells were cultured in the wells of 96-well flat-bottom microplates (Thermo Fisher Scientific) containing 100 μL of Dulbecco’s modified Eagle’s medium (Gibco) supplemented with 10% fetal bovine serum (Gibco) and 1% solution of antibiotics (penicillin and streptomycin; Gibco) at 37 °C with 5% CO_2_ and 95% humidity for 24 h. Subsequently, 100 μL of each anti-*P. insidiosum* substance double diluted with fresh medium was added to each well with the final concentrations in the wells ranging from 0.125 to 0.004 mg/mL in case of xanthyletin and from 12.5 to 0.391 mg/mL in case of both secondary metabolites from *P. stutzeri* and *K. pneumoniae*. The plates were then incubated under the same conditions. Further, the solutions in the wells were carefully discarded and cells were washed with PBS. Then, the MTT solution (0.5 mg/mL in PBS, Invitrogen) was added at an amount of 100 μL each into the wells and the mixture was kept at 37 °C with 5% CO_2_ and 95% humidity for 4 h. After removal of the supernatant, the formazan crystals were dissolved by adding 100 μL of dimethyl sulfoxide per well and the absorbance was determined at a wavelength of 570 nm. The experiment was performed in triplicate and repeated three times under the same conditions. The rate of cytotoxicity was determined using the following formula:

$$Cell\,viability\,( \% )=(absorbance\,of\,treated\,cells/absorbance\,of\,control\,cells)\times 100.$$

Additionally, the half-maximal inhibitory concentration (IC50) was measured.

## References

[1] Gaastra W (2010). Pythium insidiosum: an overview. Vet. Microbiol..

[2] De Cock AW, Mendoza L, Padhye AA, Ajello L, Kaufman L (1987). Pythium insidiosum sp. nov., the etiologic agent of pythiosis. J. Clin. Microbiol..

[3] Mendoza L, Hernandez F, Ajello L (1993). Life cycle of the human and animal oomycete pathogen Pythium insidiosum. J. Clin. Microbiol..

[4] Thianprasit M, Chaiprasert A, Imwidthaya P (1996). Human pythiosis. Curr. Top. Med. Mycol..

[5] Mendoza L, Newton JC (2005). Immunology and immunotherapy of the infections caused by Pythium insidiosum. Med. Mycol..

[6] Suthiwong J, Thongsri Y, Yenjai C (2017). A new furanocoumarin from the fruits of Scaevola taccada and antifungal activity against Pythium insidiosum. Nat. Prod. Res..

[7] Thongsri Y (2014). Detection of diketopiperazine and pyrrolnitrin, compounds with anti-Pythium insidiosum activity, in a Pseudomonas stutzeri environmental strain. Biomed. Pap. Med. Fac. Univ. Palacky. Olomouc Czech Repub..

[8] Montagner C (2008). Antifungal activity of coumarins. Z. Naturforsch C. J. Biosci..

[9] Ndendoung Tatsimo SJ, Marc Lamshöft J-D-DT, Mouafo FerdinandTalontsi, Lannang AlainMeli, Sarkar Prodipta, Prasanta Kumar Bag MS (2015). LC-MS guided isolation of antibacterial and cytotoxic constituents from Clausena anisata. Medicinal Chem. Res..

[10] Leon M (2009). Antifungal activity of selected indigenous pseudomonas and bacillus from the soybean rhizosphere. Int. J. Microbiol..

[11] Al-Rubaye AF, Hameed KM (2017). IH. Characterization of antifungal secondary metabolites produced by Klebsiella pneumoniae and screening of its chemical compounds using GC-MS. Int. J. Curr. Pharm. Rev. Res..

[12] Tracy LE, Minasian RA, Caterson EJ (2016). Extracellular Matrix and Dermal Fibroblast Function in the Healing Wound. Adv. Wound Care.

[13] Dick, M.K., Miao, J.H. & Limaiem, F. in StatPearls (Treasure Island (FL); 2020).

[14] Hilton RE, Tepedino K, Glenn CJ, Merkel KL (2016). Swamp cancer: a case of human pythiosis and review of the literature. Br. J. Dermatol..

[15] De Souza Silveira Valente, J. *et al*. *In vitro* anti-Pythium insidiosum activity of biogenic silver nanoparticles. *Med Mycol* (2018). 10.1093/mmy/myy147 30597067

[16] Loreto ES, Tondolo JSM, Santurio JM, Alves SH (2019). Screening of antibacterial drugs for antimicrobial activity against Pythium insidiosum. Med. Mycol..

[17] Araujo MJ, Bosco SM, Sforcin JM (2016). Pythium insidiosum: inhibitory effects of propolis and geopropolis on hyphal growth. Braz. J. Microbiol..

[18] De Souza Silveira Valente J (2016). *In Vitro* Activity of Melaleuca alternifolia (Tea Tree) in Its Free Oil and Nanoemulsion Formulations Against Pythium insidiosum. Mycopathologia.

[19] De Souza Silveira Valente J (2016). *In Vitro* Susceptibility of Pythium insidiosum to Melaleuca alternifolia, Mentha piperita and Origanum vulgare Essential Oils Combinations. Mycopathologia.

[20] Trolezi R (2017). Stryphnodendron adstringens and purified tannin on Pythium insidiosum: *in vitro* and *in vivo* studies. Ann. Clin. Microbiol. Antimicrob..

[21] Wittayapipath K (2019). Analysis of xanthyletin and secondary metabolites from Pseudomonas stutzeri ST1302 and Klebsiella pneumoniae ST2501 against Pythium insidiosum. BMC Microbiol..

[22] Santos JCN (2018). Thimerosal changes protein conformation and increase the rate of fibrillation in physiological conditions: Spectroscopic studies using bovine serum albumin (BSA). Int. J. Biol. Macromol..

[23] Geier DA (2015). Thimerosal: clinical, epidemiologic and biochemical studies. Clin. Chim. Acta.

[24] Montagner C (2011). *In Vitro* Cytotoxic Screening of Coumarins. Lat. Am. J. Pharm..

[25] Chaiprasert A (2009). Pythium insidiosum Thai isolates: molecular phylogenetic analysis. Asian Biomed..

[26] Mosmann T (1983). Rapid colorimetric assay for cellular growth and survival: application to proliferation and cytotoxicity assays. J. Immunol. Methods.

[27] Riss, T. L. *et al*. in Assay Guidance Manual. (eds. G.S. Sittampalam *et al*.) (Bethesda (MD); 2004).

